# Rejuvenating stem cells to restore muscle regeneration in aging

**DOI:** 10.12688/f1000research.9846.1

**Published:** 2017-01-25

**Authors:** Eyal Bengal, Eusebio Perdiguero, Antonio L. Serrano, Pura Muñoz-Cánoves

**Affiliations:** 1 Department of Biochemistry, Rappaport Faculty of Medicine, Technion-Israel Institute of Technology, Haifa 31096, Israel; 2 Cell Biology Group, Department of Experimental and Health Sciences, Universitat Pompeu Fabra (UPF), Centro de Investigación Biomédica en Red Enfermedades Neurodegenerativas (CIBERNED), Barcelona, Spain; 3 Institució Catalana de Recerca i Estudis Avançats (ICREA), Barcelona, Spain; 4 Tissue Regeneration Laboratory, Centro Nacional de Investigaciones Cardiovasculares (CNIC), Madrid, Spain

**Keywords:** adult muscle stem cells, satellite cells, muscle repair, muscle regeneration, muscle ageing, sarcopenia, satellite-cell decay, satellite cell rejuvenation

## Abstract

Adult muscle stem cells, originally called satellite cells, are essential for muscle repair and regeneration throughout life. Besides a gradual loss of mass and function, muscle aging is characterized by a decline in the repair capacity, which blunts muscle recovery after injury in elderly individuals. A major effort has been dedicated in recent years to deciphering the causes of satellite cell dysfunction in aging animals, with the ultimate goal of rejuvenating old satellite cells and improving muscle function in elderly people. This review focuses on the recently identified network of cell-intrinsic and -extrinsic factors and processes contributing to the decline of satellite cells in old animals. Some studies suggest that aging-related satellite-cell decay is mostly caused by age-associated extrinsic environmental changes that could be reversed by a “youthful environment”. Others propose a central role for cell-intrinsic mechanisms, some of which are not reversed by environmental changes. We believe that these proposals, far from being antagonistic, are complementary and that both extrinsic and intrinsic factors contribute to muscle stem cell dysfunction during aging-related regenerative decline. The low regenerative potential of old satellite cells may reflect the accumulation of deleterious changes during the life of the cell; some of these changes may be inherent (intrinsic) while others result from the systemic and local environment (extrinsic). The present challenge is to rejuvenate aged satellite cells that have undergone reversible changes to provide a possible approach to improving muscle repair in the elderly.

## Introduction

Muscle is one of the few tissues with the capacity to regenerate throughout most of our life. This capacity is gradually lost and is minimal in advanced old age. Muscle regeneration relies on a heterogeneous population of adult stem cells, known as satellite cells (SCs), which reside in a niche between the muscle sarcolemma and the basal lamina of each muscle fiber ^1– 3^. The microenvironment of the SC includes interstitial cells (such as fibro/adipogenic progenitors [FAPs] and macrophages), blood vessels, extracellular matrix proteins, and secreted factors ^4– 6^. These components help to sustain the SC population in resting muscle and their regenerative capacity in response to muscle injury through as yet largely unknown mechanisms. In healthy muscle, SCs are in a quiescent, non-proliferative state but become activated and proliferate in response to muscle injury. A subset of the proliferating cells commits to differentiation and fuses with damaged fibers, while another subset of activated SCs self-renews and re-instates quiescence, thus preserving a pool of stem cells for future regeneration ^7– 9^. Balanced fate decisions are essential for maintaining the stem-cell pool and at the same time repairing muscle damage. Muscle regeneration is compromised by perturbations in aged muscle and muscular disease states that shift the equilibrium of SCs toward myogenic commitment or self-renewal ^10^.

Quiescent SCs are characterized by the expression of several molecules, including the Paired box protein Pax7 (regarded as a definitive SC marker), and by the absence of muscle regulatory factors (MRFs) ^11– 14^. Expression analysis of quiescent SCs distinguishes them from other SC fates ^15– 17^, revealing a transcription profile that includes genes involved in the inhibition of proliferation and adhesion to the anatomical niche and others required for the metabolic demands of quiescence ^18^. Interestingly, many silent genes in the quiescent SC are marked by “active chromatin”, indicating that they are in a “poised” state, primed for fast release from quiescence to the activated state ^19, 20^. Another crucial factor for maintaining SC quiescence is Notch signaling ^21– 23^. Notch activation in quiescent SCs inhibits MyoD expression and induces Pax7 expression, which further reduces MyoD protein stability ^24, 25^. Thus, at least one role of Notch signaling is to prevent MyoD expression in the quiescent state. Interestingly, the transcription factor Forkhead box protein O3 (FoxO3), also required for quiescence re-entry during self-renewal, was recently demonstrated to induce Notch signaling by increasing the expression of Notch receptors ^26^. Therefore, the FoxO3–Notch–Pax7–MyoD axis may be one pathway regulating the quiescent state (either its maintenance or reacquisition during regeneration). However, it is clear that the maintenance of SC quiescence requires other, as-yet-uncharacterized epigenetic, transcriptional, and post-transcriptional regulators.

The activation of SCs is triggered by damage-associated molecular patterns (DAMPs), growth factors, and cytokines released by resident cells and infiltrating inflammatory cells in response to muscle injury ^27– 33^. These environmental signals induce the immediate expression of the myogenic transcription factors MyoD and Myf5, which control the transcriptional program of activated SCs ^34– 36^. Transcriptome analysis of activated SCs reveals the upregulation of genes implicated in cell-cycle progression, metabolic processes, and responses to the immune system ^16, 20^. Unlike the situation in quiescence, many genes expressed in activated SCs are associated with a repressive chromatin state that is possibly needed to restrict commitment to the myogenic fate ^20, 37– 42^. A certain proportion of SCs must self-renew to preserve stem cells for future regeneration events. Like other adult stem cells, SCs can undergo symmetric or asymmetric cell division. One daughter cell of asymmetric cell division is destined to self-renew and replenish the quiescent SC pool and the other to differentiate. The mode of cell division is dependent on many parameters, including division orientation, environmental signaling events, distribution of cellular components, and expression patterns ^43, 44^. It is becoming evident that SCs are heterogeneous and are composed of subpopulations with distinct gene expression profiles and different propensities for self-renewal and differentiation (reviewed in 45).

## The decline of satellite cells with aging

**Figure 1.**
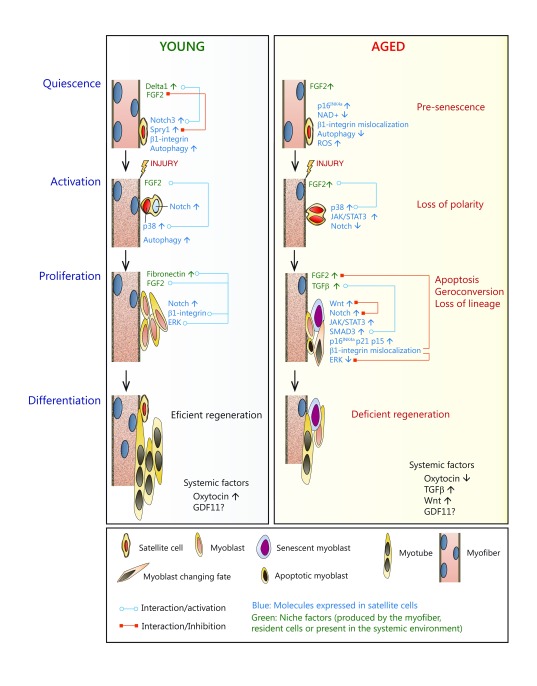
Extrinsic and intrinsic factors altering muscle stem cell regenerative functions during aging. Muscle stem cells (satellite cells), located in the basal lamina next to the myofiber, are normally in quiescence and express the Notch3 receptor, β1-integrin, and Sprouty1 (Spry1, the fibroblast growth factor 2 [FGF2] signaling inhibitor). The myofiber secretes Delta1 (a Notch ligand) to the satellite cell niche (the local microenvironment). Both Notch receptor/Delta1 interaction and β1-integrin are required for satellite cell quiescence maintenance. Quiescent satellite cells also require a sufficient amount of nicotinamide adenine dinucleotide (NAD+) to sustain mitochondrial function and fitness. As the organism ages, satellite cells as well as the systemic and niche environment undergo changes that affect the regenerative functions. The myofiber reduces the release of Delta1 and increases the production of FGF2 and transforming growth factor β (TGFβ). In the systemic circulation, during muscle damage, increased levels of TGFβ family members (with controversy on GDF11), Wnt, and oxytocin are also found, together with a reduction in the provision of fibronectin to the niche, which in turn affects the interaction with β1-integrin and FGF2-induced ERK signaling in the satellite cell, thus impacting on stem cell functions, particularly during the activation/proliferation and self-renewal stages, hence affecting the overall regeneration process. Quiescent satellite cells at old age also present elevated activity of the p38αβ mitogen-activated protein kinase (MAPK) (p38) and JAK/STAT3 signaling pathways, and, at more advanced geriatric age, the p16INK4a locus becomes derepressed. Disruption of FGF2–Spry1 signaling and delocalization of β1-integrin in old satellite cells leads to a break of quiescence, while induction of p16 ^INK4a^ at a geriatric age provokes a switch from quiescence to pre-senescence. Reduction of NAD+ in aged satellite cells is also considered a pivotal switch to induce satellite cell senescence. In response to muscle injury, young/adult muscle stem cells exit the quiescent G0 state and activate and enter the cell cycle, undergoing asymmetric division and self-renewal with induction of the p38αβ MAPK pathway in the daughter cell (due to polarized activation of fibroblast growth factor receptor 1 [FGFR1]), which will commit to the myogenic lineage and the eventual formation of new regenerated fibers. In aging muscle, p38αβ MAPK signaling is elevated in satellite cells, while FGF2 levels increase in the niche. In response to injury, the desensitized FGFR1 in old satellite cells fails to establish polarity by deregulating p38αβ signaling. As a consequence, satellite cell self-renewal is impaired in the old muscle, and an increased number of cells become committed to differentiation, with signs of apoptosis. In addition, while at a young age cells infiltrating the injured muscle produce fibronectin, which extensively occupies the niche, at old age the production of fibronectin is severely reduced, thus affecting the interaction with β1-integrin and the crosstalk with the FGF2–ERK MAPK signaling axis, which in turn impacts negatively on satellite cell proliferation. The proliferation, differentiation, and self-renewal capacities of old satellite cells are also perturbed by the JAK2/STAT3 pathway and by an imbalance in the Notch–Smad3 pathway (caused by high TGFβ levels in the niche), which leads to induction of CDK inhibitors (p15, p21, and p27) and of the Notch/Wnt pathway (the latter also promoting a switch of satellite cells towards a fibrogenic fate). At geriatric age, the regenerative pressure over G0 irreversibly arrested pre-senescent satellite cells drives their accelerated entry into full senescence (geroconversion). This process is accelerated by the reduced autophagy flux in aging satellite cells, which leads to dysfunctional mitochondria and increasing levels of reactive oxygen species (ROS), which contribute to the terminal senescent state. Altered levels of circulating factors, such as oxytocin, with aging also impact negatively on muscle regeneration (the levels of GDF11 are controverted). In summary, satellite cell intrinsic and extrinsic factors that undergo changes during aging can cooperate and synergize (or, alternatively, counteract their activities), thus altering the functions of aged satellite cells, which accounts for the deficient age-associated skeletal muscle regeneration.

Muscle aging is characterized by loss of mass and function, a process known as sarcopenia, and by a decline in repair capacity as a consequence of functional impairment and numerical reductions of SCs ^17, 46, 47^. This decline is not the cause of sarcopenia but blunts muscle recovery after injury in elderly individuals. Recent studies have addressed the potential involvement of SCs in sarcopenia, with distinct conclusions ^48, 49^; however, this review focuses only on the altered functions of SCs during the muscle regeneration process with aging. The age-related regenerative decline of SCs is due to age-associated extrinsic/environmental changes as well as cell-intrinsic/autonomous changes ( Figure 1). These intrinsic changes may have accumulated in the SC during its life and lead to reversible or irreversible intracellular damage. Aging-associated changes that reduce SC function include increased DNA damage, modifications to the epigenome and transcriptome, modified signaling pathways, damage to proteins, and altered metabolism, all of which lead to reduced proliferation and self-renewal. Damage accumulation can lead to a “point of no return” of the very old (geriatric) SC that enters a pre-senescent state or undergoes apoptosis ^47^.

### Intrinsic changes

Aging SCs show evidence of several cell-intrinsic changes that are likely interconnected, including genomic instability, DNA damage, oxidative damage, and deteriorated mitochondrial function. Compared with their progeny, SCs are relatively resistant to DNA damage ^50^. However, because of their low turnover, SCs cannot dilute the accumulation of DNA damage resulting from a lifetime of genotoxic stress exposure and gradual loss of antioxidant capacity ^51^. DNA damage accumulation may be involved in many or all of the dysregulated processes affecting SCs, including cell-cycle division, proteostasis, senescence, and cell death. The effects of DNA damage on other dysregulated processes are yet to be determined.

The age-associated functional defects observed in SCs may reflect alterations to epigenetic and transcriptional programs. Transcriptional changes could explain reduced antioxidant activity, changes in protein folding, reduced myogenic differentiation, and the tendency of these cells to adopt fibroblastic and adipogenic fates ^52^. The altered transcriptional program could be due in part to dramatic changes occurring in the epigenetic landscape ^20^, which include changes in the DNA methylation pattern and post-translational histone modifications. Recent studies demonstrate a progressive increase in DNA methylation in aging muscle ^53– 55^. In general, *de novo* DNA methylation of CpG islands recruits polycomb repressive complex 2 (PRC2) to gene promoters in aged cells, and SCs isolated from aged mice show elevated levels and altered distribution of the H3K27me3 repressive mark ^20^. These changes likely affect gene expression and contribute to the deregulation of signaling pathways necessary for an efficient regenerative response, as described above. One pathway that is highly active in aged SCs is the p38 mitogen-activated protein kinase (MAPK) (reviewed in ^56– 58^). It remains unclear if high p38 MAPK activity in SCs is induced by intracellular signal transduction/transcriptional changes (intrinsic) or by extracellular ligands (extrinsic). High p38 MAPK activity is reported to reduce proliferative activity ^59^ and to decrease asymmetric cell divisions ^60^, ultimately reducing the number of self-renewed SCs. Self-renewal and regenerative capacity of “old SCs” is restored by *ex vivo* treatment with a small-molecule p38 MAPK inhibitor ^44^. Another gene whose expression is affected by epigenetic changes is *Cdkn2a*, which encodes the cell-cycle inhibitor p16 ^INK4A^, thought to drive cellular senescence ^34^. In young SCs, p16 ^INK4A^ is silenced by the PRC1-mediated repressive histone H2AK119Ub modification; H2AK119Ub is significantly reduced in SCs isolated from geriatric mice, resulting in p16 ^INK4A^ derepression ^47^. Increased p16 ^INK4A^ levels cause geriatric SCs to enter a pre-senescent state. Interestingly, p38 MAPK may induce cellular senescence by activating p16 ^INK4A^ ^61^. A model could thus be drawn in which intrinsic p38 activity affects old SCs in at least two ways: reducing asymmetric cell division and self-renewal and also activating p16 ^INK4A^ expression, driving these cells to a pre-senescent state.

The SCs of old mice also have elevated activity of the JAK–STAT pathway ^62, 63^. STAT3 drives the expression of MyoD and commitment to myogenic differentiation, and its high activity therefore reduces SC self-renewal. As with p38 MAPK, transient pharmacological inhibition of STAT3 in aged mice increases the population of proliferating SCs and improves muscle regeneration ^63^.

Another cell-intrinsic change observed in old and geriatric SCs is unbalanced proteostasis (protein homeostasis) ^64^. SCs from geriatric mice are characterized by low baseline autophagy (a quality-control mechanism whereby intracellular proteins and organelles are degraded within the lysosome), resulting in accumulation of damaged proteins, dysfunctional mitochondria, and oxidative stress that lead to the senescent state ^64^. Consistent with this, SC senescence in aging mice is driven by a decline in the level of oxidized cellular nicotinamide adenine dinucleotide (NAD+) that impairs mitochondrial activity. Treatment with the NAD+ precursor nicotinamide riboside rejuvenates SC function ^65^. It is presently unknown whether the block in autophagy and mitochondrial function is linked to the activation of p38 MAPK, and there is a need for more research into the potential links between proteotoxicity and senescence in aging stem cells.

### Extrinsic changes

SCs are affected by the local microenvironment (niche) as well as the systemic circulation, both of which undergo aging-associated alterations. The expression of several extracellular ligands increases during aging in the niche, compromising SC quiescence and reducing their regenerative potential. Niche FGF signaling is elevated with aging owing to the release of FGF2 by myofibers and decreased expression of Spry1, which encodes Sprouty1, an inhibitor of FGF signaling. Genetic elimination of Spry1 in SCs promotes FGF signaling, resulting in loss of quiescence and a subsequent reduction in SC number. Spry1 elimination during adult muscle repair led to persistent ERK MAPK activation, which impaired the self-renewal of a subset of SCs ^66^. In contrast to its detrimental role for SC quiescence maintenance, FGF signaling plays an important role in SC proliferation *in vitro* and *in vivo* ^67, 68^, thus suggesting a possible dual role for some growth factors during the regeneration stages. Other signaling molecules showing increased expression in the aging niche include TGFβ and canonical Wnt, both implicated in the suppression of SC stemness and in their transdifferentiation from a myogenic to a fibrogenic lineage ^52, 69^. It is worth noting that the transdifferentiation of SCs into other cell types, such as fibroblastic or adipogenic cells, may constitute rather infrequent events in aging or dystrophic muscle and in cell culture ^52, 69– 72^. Notch signaling, required to maintain the quiescent state, is reduced in the aged niche, and the importance of Notch in maintaining regenerative potential is demonstrated by the finding that Notch inhibition in young SCs causes regenerative defects while its activation in aged SCs restores their regeneration capacity ^73– 75^. Moreover, old myofibers express insufficient amounts of the Notch ligand Delta1, which is necessary to maintain SC quiescence ^76^. Recently, additional evidence on the requirement of SC–niche interactions for the maintenance of SC function and tissue repair capacity has been provided ^77, 78^. The expression of the cell surface receptor β1-integrin and the extracellular matrix (ECM) protein fibronectin is altered in old SCs and their niche, respectively ^79, 80^. Importantly, restoring their function rescues muscle regeneration in old mice. How these various local signals interconnect awaits further investigation.

The influence of the systemic circulation on SCs was demonstrated in heterochronic whole muscle transplant experiments ^81– 85^ and heterochronic parabiosis, wherein two mice are surgically joined such that they share the same circulatory system ^74, 86– 89^. Interestingly, joining young and aged mice improved the regenerative response to muscle injury in the aged partner ^73, 74^, indicating that young blood contains “rejuvenating factors”, and a major effort has been directed at identifying these molecules. One candidate is oxytocin, a hypothalamic hormone that declines with age in the blood and whose receptor is downregulated in SCs of aged mice ^90^. Administration of oxytocin to aged mice enhances SC proliferation and differentiation and improves overall regenerative potential after muscle injury ^90^. Another candidate is GDF11; however, its influence on SCs is debated. GDF11 is a member of the TGFβ family that shows structural and functional homology to myostatin ^91^. While one group observed its decline in the blood of aged animals and humans and showed that administration of recombinant GDF11 to old mice improved SC regeneration ^89^, another group reported that the levels of GDF11 increase with age and that its administration to old mice has no beneficial effects and may even worsen regeneration after muscle injury in young mice ^92^. More recent studies found no evidence that GDF11 rejuvenates old stem cells or extends lifespan in models of progeria and reported no improvement in muscular dystrophy ^93– 96^.

Distinct cell types residing in the niche or infiltrating the injured muscle have been shown to influence SC functions by releasing growth factors and cytokines, which may act at the distinct myogenic stages during the regeneration process. These cell types include FAPs and other resident progenitor cells, several immune cell types such as macrophages, eosinophils, and T lymphocytes, neurons, or endothelial cells ^97– 110^. Because these cells may also experience age-related alterations, it is likely that the crosstalk between them and the SCs will be affected with aging and hence provoke consequences on the repair process. Similarly, changes in the interactions between cells and the ECM during aging, by modifying tissue stiffness and topography, may alter SC regenerative functions ^6, 78– 80, 111, 112^.

## Can SC function be restored in aged individuals?

The significant advances in the understanding of SC aging open up real possibilities for improving SC regenerative potential as a possible treatment for aging and diseased muscles. The emerging evidence indicates that the functional and numerical loss of SCs is a progressive process occurring throughout the lifetime of the organism. The long-lived quiescent SC accumulates many lesions caused by loss of homeostasis, metabolic alterations, and the aging environment. Although this process is gradual, it is accelerated in advanced old age to the extent that SCs become practically non-functional owing to senescence or apoptosis. In this context, disputes about which factors, intrinsic or extrinsic, are more dominant in dictating the fate of old SCs seem misplaced, and it is likely that both make important contributions to SC functional decline with aging. A degree of success has been obtained in restoring the regenerative capacity of old muscle with both parabiosis experiments (extrinsic effect) and transplantation of *ex vivo*-rejuvenated SCs into old animals (intrinsic effect). The simplest explanation for these effects is the heterogeneous nature of SCs. Even in old age, the SC population includes a small percentage of functional SCs, with only limited accumulated damage that can be reversed still by extrinsic signaling factors or by *ex vivo* pharmacological inhibition of stress pathways such as p38 MAPK or JAK/STAT3. It is thus likely that the success of biochemical or genetic strategies applied to old SCs in transplantation experiments results from the proliferative amplification of a subset of highly regenerative cells. Alternatively, the health and fitness of old SCs could be increased by refueling “clean up” activities such as autophagy (which declines with aging) to eliminate damage, thus improving SC regenerative capacity after muscle injury and in transplantation procedures. Future interventions that could also be considered for combating age-related muscle regenerative decline may utilize the restoration of SC–niche interactions via the delivery of bioengineered molecules.

The accumulated evidence outlined in this review indicates a number of clear directions for future research. The key finding that the SC pool enters a state of irreversible senescence at a geriatric age ^47^ implies that any treatment to rejuvenate endogenous stem cells should be implemented before this point of no return. It is also important to consider the link between SC regenerative potential and quiescence. It is generally well accepted that the more quiescent a stem cell is, the more regenerative capacity it has. It has also become clear that somatic stem cell populations are heterogeneous, with cells showing differing levels of quiescence ^113^. Subpopulations of quiescent SCs with distinct regenerative capacities have been identified based on the differential expression of markers such as Pax7, CD34, Myf5, and M-Cadherin ^13, 114– 117^. Highly quiescent subpopulations probably change with aging to become less quiescent and therefore of reduced regenerative capacity. SC heterogeneity should therefore be further investigated, with the aim of deciphering the molecular basis of quiescence. Understanding the quiescent state will allow early intervention aimed at preserving the highly regenerative quiescent subpopulations throughout life. Likewise, strategies directed towards the expansion of relevant subpopulations of resident progenitor cells in the SC niche may be envisioned for reversing the age-associated muscle regenerative loss. Another unresolved issue is the interaction among the various events contributing to the loss of SC regenerative potential with aging. Research needs to focus on determining which events are causative and which are consequential. For example, DNA damage may induce the loss of baseline autophagy flux in old SCs, or alternatively DNA damage may be the consequence of oxidative stress resulting from the loss of autophagy flux. Defining the hierarchy of events leading to SC deterioration will enable the targeting of upstream events in order to achieve more efficient rejuvenation of SCs. Last but not least, in a low-turnover tissue like muscle, much of the damage to the quiescent SC is the result of the gradual decline (aging) of the niche composition and the systemic system. Future efforts to rejuvenate the regenerative potential of SCs should thus adopt a holistic view of the SC and its supportive environment.

Current efforts to rejuvenate SCs in aged mice include genetic and pharmacological inhibition of p16 ^INK4a^ ^47^, STAT3 ^62, 78^, and p38 MAPK ^59^, augmentation of autophagic flux ^64^, NAD+ repletion ^65^, and the administration of rejuvenating hormones like oxytocin ^90^. While these approaches hold great promise, their translation from mouse to human will require significant technological advances to eliminate or minimize the potentially broad side effects. Interestingly, SC activity has been found to increase in response to simple lifestyle changes that modify cell metabolism, such as adopting a low-calorie diet ^118^. Similarly, exercise has been shown to enhance SC numbers and function and hence promote better muscle regeneration in rodents ^119– 122^. This serves as a reminder that we should consider not only sophisticated methods but also simple innovative approaches deriving from our understanding of the system.

## Abbreviations

ECM, extracellular matrix; FAP, fibro/adipogenic progenitor; MAPK, mitogen-activated protein kinase; MRF, muscle regulatory factor; NAD+, nicotinamide adenine dinucleotide; SC, satellite cell.
